# Successful Management of Suspected Epstein–Barr Virus‐Associated Hemophagocytic Lymphohistiocytosis in BRAF‐Mutant Cholangiocarcinoma Following Treatment With Immune Checkpoint Inhibitors and BRAF/MEK Inhibitors: A Case Report

**DOI:** 10.1002/cnr2.70504

**Published:** 2026-02-28

**Authors:** Akira Shibata, Michihiro Ono, Shutaro Oiwa, Atsushi Uesugi, Seiya Saito, Makoto Usami, Tomoyuki Abe, Masahiro Yoshida, Masahiro Maeda, Kohichi Takada

**Affiliations:** ^1^ Department of Residency and Clinical Training Steel Memorial Muroran Hospital Hokkaido Japan; ^2^ Department of Gastroenterological Surgery II Hokkaido University Faculty of Medicine Hokkaido Japan; ^3^ Department of Pancreatobiliary Medicine Steel Memorial Muroran Hospital Hokkaido Japan; ^4^ Department of Gastroenterology Steel Memorial Muroran Hospital Hokkaido Japan; ^5^ Division of Medical Oncology, Department of Internal Medicine Sapporo Medical University School of Medicine Japan; ^6^ Department of Medical Oncology Steel Memorial Muroran Hospital Hokkaido Japan; ^7^ Department of Hematology Steel Memorial Muroran Hospital Hokkaido Japan

**Keywords:** BRAF/MEK inhibitor, cholangiocarcinoma, Epstein–Barr virus, hemophagocytic lymphohistiocytosis, immune checkpoint inhibitor

## Abstract

**Background:**

The BRAF V600E mutation is a rare genetic alteration in cholangiocarcinoma for which sequential therapy with immune checkpoint inhibitors (ICIs) and BRAF/MEK inhibitors may be effective. However, the full spectrum of adverse events associated with the sequential use of these agents remains unclear. Hemophagocytic lymphohistiocytosis (HLH) is a fatal cytokine release syndrome that can be triggered by a cytokine storm.

**Case:**

A 49‐year‐old man underwent left lateral hepatectomy and adjuvant chemotherapy for cholangiocarcinoma and subsequently developed lymph node recurrence. The patient initially received systemic chemotherapy with gemcitabine, cisplatin, and S‐1; however, due to disease progression, the regimen was switched to gemcitabine, cisplatin, and durvalumab. After these treatments, a BRAF V600E mutation was identified through comprehensive gene panel testing, leading to the initiation of BRAF and MEK inhibitors. However, 3 months later, the patient presented to the emergency department of Steel Memorial Muroran Hospital with fever and fatigue in June 2024. He was initially diagnosed with septic shock but was unresponsive to broad‐spectrum antibiotics. Laboratory tests revealed elevated ferritin levels, elevated soluble interleukin‐2 receptor levels, and Epstein–Barr Virus (EBV)‐DNA. HLH was diagnosed, and multidisciplinary treatment, including steroid pulse therapy, was initiated. The patient's condition improved dramatically, and he survived.

**Conclusion:**

We report a rare case of cholangiocarcinoma with suspected EBV‐associated HLH that developed after sequential therapy with an ICI and BRAF/MEK inhibitors. Clinicians should consider HLH as a differential diagnosis in patients with a history of ICI therapy who present with severe unexplained inflammation or shock. Prompt diagnosis and multidisciplinary management are crucial to prevent death.

## 1 Introduction

Cholangiocarcinoma (CCA), a malignancy arising from the biliary epithelium, is anatomically classified into intrahepatic, perihilar, and distal types. Although the prognosis for patients with unresectable or metastatic CCA remains poor, in recent years, advances in the genomic analysis of CCA have identified several actionable genetic alterations. For example, the BRAF V600E mutation is a rare driver mutation found in approximately 5% of CCA cases [1, 2]. Combination therapy with a BRAF inhibitor and MEK inhibitor has shown efficacy in patients with BRAF V600E‐mutant unresectable or metastatic CCA [3].

The standard first‐line treatment for unresectable CCA is a regimen combining gemcitabine and cisplatin with an immune checkpoint inhibitor (ICI) [4, 5]. Patients with BRAF V600E‐mutant CCA are anticipated to receive second‐line therapy with BRAF/MEK inhibitors [3, 6]. However, the spectrum of adverse events (AEs) associated with the sequential administration of ICIs and molecularly targeted drugs is not yet fully understood.

Hemophagocytic lymphohistiocytosis (HLH), a life‐threatening syndrome characterized by severe systemic inflammation resulting from excessive cytokine production, can be triggered by various underlying conditions, including malignancies, autoimmune diseases, and infections, particularly Epstein–Barr virus (EBV) infection [7]. Recently, there has been an increase in reports of HLH as an immune‐related AE (irAE) [8]. Herein, we report a rare case of CCA complicated by HLH, in which an ICI, BRAF/MEK inhibitors, and EBV reactivation were all considered potential contributing factors.

## 2 Case

A 49‐year‐old man underwent a left lateral hepatectomy for intrahepatic CCA at Steel Memorial Muroran Hospital in April 2020; however, para‐aortic lymph node metastases developed 1 year later in July 2021. In response, the patient received 19 courses of a triple chemotherapy regimen comprising gemcitabine (1000 mg/m^2^), cisplatin (25 mg/m^2^), and S‐1 (80 mg/m^2^), administered every 3 weeks from August 2021. Three years later in May 2023, as the disease progressed, the patient received eight courses of a regimen consisting of gemcitabine (1000 mg/m^2^), cisplatin (25 mg/m^2^), and durvalumab (1500 mg), followed by seven courses of durvalumab (1500 mg) monotherapy as maintenance therapy from October 2023. During durvalumab treatment, he developed adrenal insufficiency as an irAE and was administered hydrocortisone replacement therapy.

Six months after starting durvalumab maintenance therapy in April 2024, re‐enlargement of the lymph nodes was observed. Comprehensive genomic profiling using FoundationOne CDx assay (Foundation Medicine Inc., Cambridge, MA, USA) identified a BRAF V600E mutation. Based on this, second‐line therapy with dabrafenib (150 mg twice daily), a BRAF inhibitor, and trametinib (2 mg once daily), a MEK inhibitor, was initiated. Fever and fatigue consistently developed 1 week after each treatment initiation and resolved upon temporary discontinuation.

Three months after the initiation of the BRAF/MEK inhibitors, the patient presented to the emergency department of Steel Memorial Muroran Hospital in June 2024, with a 3‐day history of fever and malaise. On arrival, his body temperature was 40.0°C, and his blood pressure was 87/60 mmHg. Physical examination revealed no other remarkable findings. Laboratory tests showed a white blood cell count of 4.11 × 10^3^/μL, hemoglobin level of 13.7 g/dL, and platelet count of 107 × 10^3^/μL. Biochemical tests revealed the following abnormalities: lactate dehydrogenase (LDH), 336 U/L (reference: 124–222 U/L); creatinine, 1.69 mg/dL (reference: 0.65–1.07 mg/dL), and C‐reactive protein (CRP), 21.1 mg/dL (reference: < 0.3 mg/dL). Coagulation tests also revealed abnormal results, with a prothrombin time‐international normalized ratio of 1.20 (reference: 0.85–1.15) and fibrin/fibrinogen degradation product level of 36.5 μg/mL (reference: < 5 μg/mL), meeting the diagnostic criteria for disseminated intravascular coagulation (DIC). A computed tomography (CT) scan of the chest, abdomen, and pelvis showed no obvious source of infection.

**FIGURE 1:**
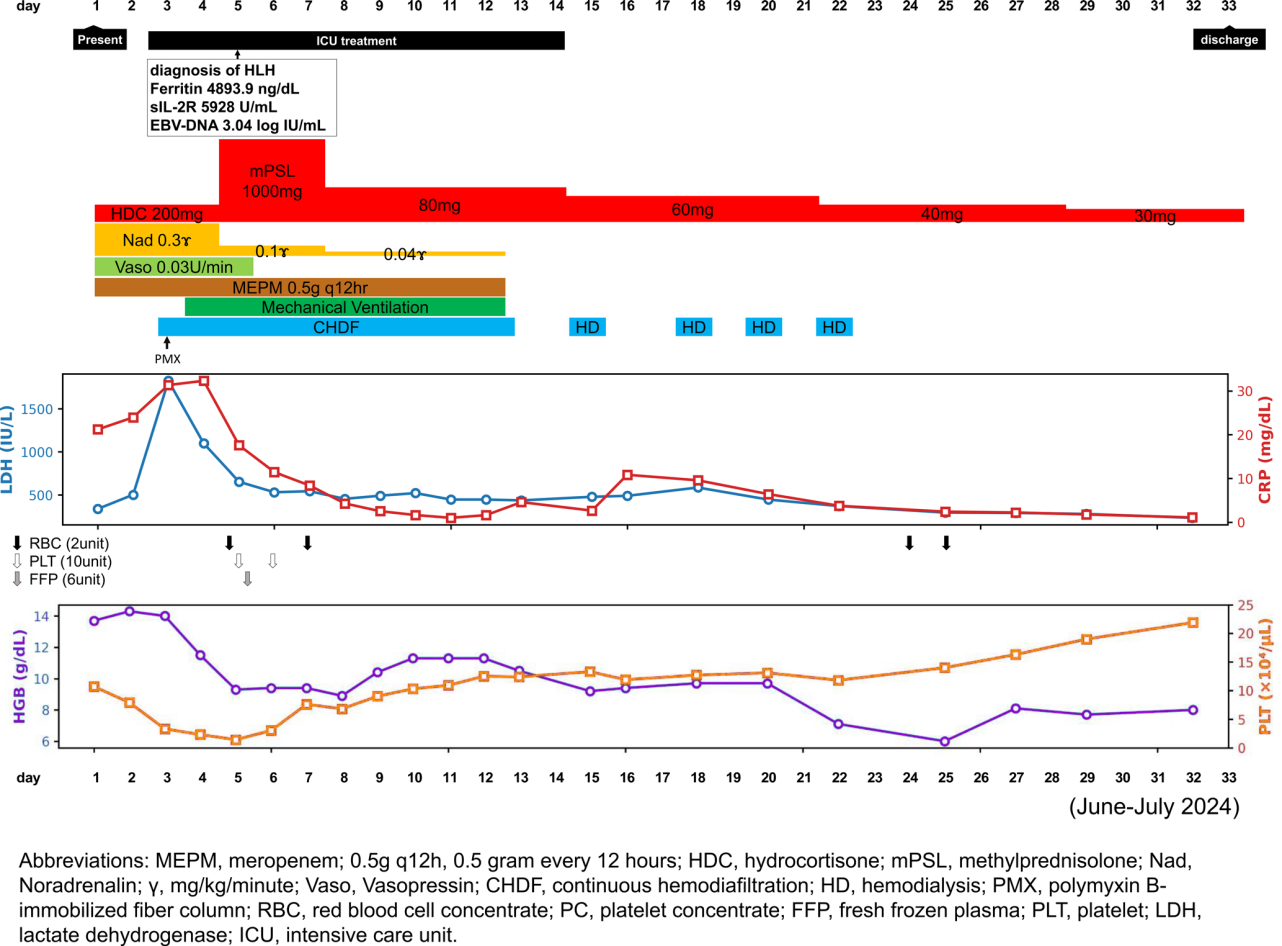
Clinical course and laboratory findings during steroid therapy. The graph illustrates the clinical course following the initiation of high‐dose methylprednisolone pulse therapy (1000 mg/day for 3 days), with subsequent tapering. After commencing steroid pulse therapy, serum lactate dehydrogenase (LDH) levels progressively decreased, and the declining trend in platelet counts was halted. Concurrently, both blood pressure and respiratory status improved, allowing withdrawal of vasopressors and mechanical ventilation. The upper graph shows trends in LDH (IU/L) and C‐reactive protein (CRP, mg/dL), while the lower graph displays hemoglobin (Hb, g/dL) and platelet (PLT, × 10^4^/μL) counts. Blood transfusions, continuous hemodiafiltration (CHDF), hemodialysis (HD), and antimicrobial treatments are also annotated along the timeline. MEPM, meropenem; 0.5 g q12h, 0.5 g every 12 h; HDC, hydrocortisone; mPSL, methylprednisolone; Nad, Noradrenalin; γ, mg/kg/min; Vaso, Vasopressin; CHDF, continuous hemodiafiltration; HD, hemodialysis; PMX, polymyxin B‐immobilized fiber column; RBC, red blood cell concentrate; PC, platelet concentrate; FFP, fresh frozen plasma; PLT, platelet; LDH, lactate dehydrogenase; ICU, intensive care unit.

The patient was initially diagnosed with septic shock and DIC; thus, multidisciplinary treatment with broad‐spectrum antibiotics (meropenem 1 g every 12 h), fluid resuscitation, and vasopressors (norepinephrine) and hydrocortisone (200 mg/day for 4 days) for severe sepsis was started (Figure 1). However, his condition worsened by Day 3, with progressive fluid retention leading to respiratory and renal function. He was also oliguric, with a daily urine output of approximately 200 mL, and had a persistent high fever of over 39°C. Laboratory findings showed persistent thrombocytopenia and continued elevation of CRP and LDH levels. Norepinephrine was increased to the maximum dose, and vasopressin was added.

As his condition became too difficult to manage in the general ward, the patient was transferred to the intensive care unit (ICU), where direct hemoperfusion with a polymyxin B‐immobilized fiber column and continuous hemodiafiltration (CHDF) were initiated. On day 4, he developed progressive acute respiratory failure, requiring tracheal intubation and mechanical ventilation. On Day 5, owing to progressive bicytopenia due to anemia and thrombocytopenia and worsening coagulopathy, he was transfused with red blood cells, platelets, and fresh frozen plasma. Laboratory values at that time were as follows: white blood cell count, 16.1 × 10^3^/μL; hemoglobin level, 8.9 g/dL; and platelet count, 14 × 10^3^/μL (Table 1). Owing to a persistently marked elevation of LDH level, HLH was considered in the differential diagnosis. Additional laboratory tests revealed a triglyceride level of 224 mg/dL (reference: 36–162 mg/dL), a ferritin level of 4893 ng/mL (reference: 30–400 ng/mL), and a soluble interleukin‐2 receptor (sIL‐2R) level of 5928 U/mL (reference: 145–519 U/mL). Furthermore, plasma EBV DNA was detected at 3.04 × 10^5^ copies/mL (Table 1). Moreover, the patient was positive for EBV nuclear antigen (EBNA), suggesting EBV reactivation rather than primary infection. As the patient's critical condition necessitated mechanical ventilation, he could not be placed in the prone position required for bone marrow aspiration; thus, hemophagocytosis could not be confirmed. Nevertheless, he met four of the eight HLH‐2004 criteria [9]: fever, bicytopenia (anemia and thrombocytopenia), hyperferritinemia, and high sIL‐2R levels, leading to a clinical diagnosis of HLH. Furthermore, the HScore was calculated as 199 using the online calculator (https://saintantoine.aphp.fr/score/), consistent with a high probability of HLH according to Fardet et al.'s criteria [10]. Thus, steroid pulse therapy (methylprednisolone 1 g/day for 3 days) was initiated on the same day, following which, prednisolone was started at 80 mg/day and tapered by 20 mg every week. Tocilizumab, etoposide, and cyclosporine were not administered because the patient showed a rapid clinical response to steroid pulse therapy alone.

**TABLE 1:** Laboratory findings during the clinical course.

Laboratory parameters	At presentation	Day 5(HLH diagnosis)	Day 7(48 h after steroid administration)	Reference ranges
White blood cells, ×10^3^/μL	4.11	16.10	8.54	3.8–8.0
Hemoglobin, g/dL	13.7	8.9	9.4	14.0–17.5
Platelets, ×10^3^/μL	107	14	76	100–330
AST, U/mL	47	195	62	1–38
ALT, U/mL	24	153	84	2–40
Creatinine, mg/dL	1.69	6.02	4.69	0.65–1.07
Total bilirubin, mg/dL	0.6	2.4	0.9	0.1–1.0
LDH, U/L	336	652	542	124–222
CRP, mg/dL	21.18	17.56	8.39	< 0.3
PT‐INR	1.2	1.19	0.94	0.85–1.15
FDP, μg/mL	36.5	229.7	36.5	< 5
Fibrinogen, mg/dL	438.3	161.2		158–400
Ferritin, ng/dL		4893.9		39.4–340
Triglycerides, mg/dL		224		36–162
sIL‐2R (CD25), U/mL		5928		145–519
EBV‐DNA, log IU/mL		3.04		< 0

*Note:* Laboratory values at presentation, on Day 5 at the time of hemophagocytic lymphohistiocytosis (HLH) diagnosis, and on day 7 (48 h after initiation of methylprednisolone pulse therapy). Markedly elevated ferritin (4893.9 ng/dL) and soluble IL‐2 receptor (sIL‐2R, 5928 U/mL) levels on Day 5 supported the diagnosis of HLH. Following steroid pulse therapy, the blood cell counts improved, platelet count recovered from 14 to 76 k/μL, and white blood cell count stabilized.

Abbreviations: ALT, alanine transaminase; AST, aspartate aminotransferase; CRP, C‐reactive protein; EBV, Epstein–Barr virus; FDP, fibrin/fibrinogen degradation products; HLH, hemophagocytic lymphohistiocytosis; LDH, lactate dehydrogenase; PT‐INR, prothrombin time‐international normalized ratio; sIL‐2R, soluble interleukin‐2 receptor.

Following the initiation of steroid pulse therapy, the patient's general condition improved. His blood counts recovered, and his vital signs stabilized, with laboratory tests showing a white blood cell count of 8.54 × 10^3^/μL, hemoglobin level of 9.4 g/dL, and platelet count of 76 × 10^3^/μL on Day 7 (Table 1). The patient was successfully weaned from mechanical ventilation and taken off CHDF. He was transferred back to the general ward on Day 14 and discharged on day 33 (Figure 1). Post‐discharge CT and positron emission tomography‐CT scans revealed new metastases in the sacrum and lungs. He received radiation therapy and denosumab for bone metastases. After receiving 17 courses of mFOLFOX6 chemotherapy, he achieved a complete response (CR) and is currently being followed up without any ongoing treatment as of December 2025.

Durvalumab and BRAF/MEK inhibitors were permanently discontinued due to the severity of the adverse event. The patient provided verbal informed consent for publication of this report.

## 3 Discussion

We report a rare case of CCA with HLH that developed after sequential therapy with durvalumab and BRAF/MEK inhibitors. The clinical course of this case mimicked septic shock, which made the diagnosis challenging. Prompt diagnosis and multidisciplinary therapeutic intervention were essential for saving the patient's life.

HLH is caused by the excessive activation of cytotoxic T cells and macrophages, followed by a cytokine storm [11]. The clinical symptoms are non‐specific and include persistent fever, hepatosplenomegaly, and cytopenia. A delay in diagnosis can lead to multi‐organ failure and a fatal outcome [7]. The initial clinical presentation of our patient was difficult to distinguish from severe sepsis and DIC. However, the lack of response to broad‐spectrum antibiotics and persistently elevated LDH levels raised the suspicion of HLH; hyperferritinemia and high sIL‐2R levels ultimately led to the clinical diagnosis.

The pathogenesis of HLH in this case was likely multifactorial. First, immune dysregulation due to prior durvalumab therapy was a possible contributor. Although ICIs exert their anti‐tumor effects by inhibiting the negative regulation of T cells, they can also trigger excessive T‐cell activation, leading to HLH as an irAE [8]. Our patient had a history of adrenal insufficiency as an irAE during durvalumab treatment, suggesting an immunological susceptibility to ICIs. Second, the direct or indirect involvement of the BRAF/MEK inhibitors should be considered. HLH is a rare AE associated with BRAF/MEK inhibitors, with only a few cases reported in patients with malignant melanoma [12, 13, 14, 15, 16]. In our case, the patient experienced recurrent fever after the initiation of BRAF/MEK inhibitors. As fever is a common AE of BRAF/MEK inhibitors [17], it was initially managed with antipyretics. However, we hypothesize that the drug‐induced systemic inflammation, potentially acting as a “second hit” in an immune system already primed by ICIs, may have escalated into a fulminant cytokine storm. This remains a hypothetical mechanism, and further research is needed to clarify the immunopathological interactions between ICIs and subsequent inflammatory triggers, such as BRAF/MEK inhibitors, in the development of HLH. Third, the reactivation of EBV could have been a factor. Although EBV remains latent in B cells and is normally controlled by CD8^+^ cytotoxic T lymphocytes, it is a potent trigger for HLH, often reactivating in the context of immunosuppression in patients with cancer [18]. ICIs are expected to enhance the immune response against EBV infection by restoring T cell function [19]. However, in this patient, the preceding ICI therapy may have altered the immune environment and T cell function. This altered immunological state, combined with immunosuppression from the underlying malignancy and prior chemotherapy, likely created a fertile ground for the reactivation of latent EBV. This case suggests that prior ICI therapy may have exacerbated the severity of EBV infection. Recent studies have reported that ICI treatment can potentiate hyperinflammatory responses in patients with viral infections, such as COVID‐19, leading to cytokine release syndrome [20, 21]. It is hypothesized that ICIs, by invigorating T‐cell responses, may lower the threshold for developing a cytokine storm when the immune system encounters a viral antigen. In our case, although the patient tested negative for COVID‐19, a similar mechanism involving EBV reactivation likely contributed to the pathogenesis of HLH.

The HLH‐2004 protocol recommends dexamethasone, etoposide, and cyclophosphamide as key therapeutic agents [22]. However, in the present case, impaired renal function posed a significant concern, making the use of etoposide and cyclophosphamide less feasible. Contrarily, previous reports have indicated the effectiveness of pulse steroid therapy in secondary HLH [9]. Based on this evidence, we opted for high‐dose corticosteroid therapy, which fortunately proved effective in this patient. The use of tocilizumab, an interleukin‐6 receptor antagonist, has also been reported as a potential treatment option [23]. In our case, tocilizumab was considered as a secondary option if the steroid pulse therapy failed to elicit a sufficient response.

This case offers significant clinical lessons. First, HLH should always be included in the differential diagnosis when a patient with a history of ICI therapy presents with a severe inflammatory condition of unknown origin during subsequent treatment, including with targeted therapies. Second, when septic shock is suspected but unresponsive to broad‐spectrum antibiotics, HLH should be considered, and relevant markers, such as triglycerides, ferritin, sIL‐2R, and EBV DNA, should be promptly measured. Third, once HLH is diagnosed, prompt multidisciplinary therapeutic intervention, including steroid‐centered immunosuppressive therapy, is essential.

We report a rare case of CCA complicated by HLH, which developed following sequential treatment with an ICI and BRAF/MEK inhibitors. To the best of our knowledge, this is the first reported case of HLH occurring after such a sequential treatment approach, regardless of the underlying malignancy. The most significant challenge in this case was distinguishing HLH from septic shock and identifying the causative factors among multiple agents. While HLH induced by BRAF/MEK inhibitors has been reported in patients with melanoma [8, 12, 13, 14, 15], our study is distinct in identifying this complication in cholangiocarcinoma and following sequential ICI therapy. Unlike previous cases where HLH resolved with drug discontinuation alone, our patient required intensive immunosuppression, highlighting the potential synergistic toxicity of sequential therapy. This case highlights that unexpected and severe irAEs can occur during modern cancer therapy, which combines drugs with different mechanisms of action. Clinicians must be aware of this potentially fatal complication and prepared to make a prompt diagnosis and initiate multidisciplinary treatment. However, this report had some limitations. First, its nature as a single‐case report and the lack of confirmation of hemophagocytosis during bone marrow examination should be considered. Second, detailed EBV serology (e.g., VCA‐IgM/IgG) and lymphocyte subset analysis were not completely assessed, which limits our ability to definitively prove primary infection versus reactivation.

## Author Contributions

All authors contributed to the conception and design of this study. Akira Shibata, Michihiro Ono, Shutaro Oiwa, Atsushi Uesugi, Seiya Saito, Makoto Usami, Tomoyuki Abe, and Masahiro Yoshida were primarily responsible for the clinical management of the patient. Michihiro Ono and Kohichi Takada conceptualized the case report. Akira Shibata and Michihiro Ono conducted the literature review and drafted the manuscript. Masahiro Maeda and Kohichi Takada contributed to the critical revision of the manuscript for important intellectual content. All authors read and approved the final version of the manuscript.

## Funding

The authors have nothing to report.

## Ethics Statement

This case report was determined not to require approval by the institutional review board, as it describes a single anonymized case in the context of routine clinical care. Verbal informed consent for publication of case details was obtained from the patient and documented in the medical record.

## Conflicts of Interest

Dr. Michihiro Ono received a research grant from the Ito Medical Science Foundation and honoraria for lectures from Daiichi Sankyo, Boston Scientific Japan, Medicos Hirata, Asahi Intecc, and AbbVie GK, outside of the submitted work. Dr. Kohichi Takada received honoraria for lectures from Daiichi Sankyo, Chugai, Eisai, Janssen, Ono, Eli Lilly, Otsuka, Sanofi, MSD, Takeda, Zeria, and Sysmex, outside of the submitted work. The other authors declare no conflicts of interest for this article. The funding source had no role in the design, practice, or analysis of this study.

## Data Availability

All data supporting the findings of this case report are available within the article.
